# 33.2 CAN THE STIGMATIZING RISKS OF THE ‘AT-RISK’ STATE BE REDUCED BY RELABELING IT ‘HIGH-RISK HEALTH’? PROMISING PILOT RESULTS FROM TWO EXPERIMENTAL VIGNETTE STUDIES AMONG THE GENERAL POPULATION AND MENTAL HEALTH PROFESSIONALS.

**DOI:** 10.1093/schbul/sby014.138

**Published:** 2018-04-01

**Authors:** Dan Koren, Shulamit Radin-Gilboa, Yulia Libas, Dana Carmi

**Affiliations:** University of Haifa

## Abstract

**Background:**

While there is a wide consensus regarding the potential benefits that early detection and intervention in clinical high-risk (CHR) states for psychosis might offer, application of this paradigm in current mental healthcare systems frequently involves concerns about the iatrogenic impact of stigma on patients, families, institutions, and the society at large. Based on examples from other areas in medicine (e.g., ‘high-risk pregnancy’ as opposed to ‘miscarriage risk syndrome’, or ‘hearing loss’ as opposed to ‘attenuated deafness’) we have recently hypothesized that restructuring CHR for psychosis states as high-risk states for universal functions (e.g., reality-testing) has the potential to reduce these concerns. The goal of this presentation is to introduce this notion and present pilot data that provide preliminary support for its validity.

**Methods:**

In the first study, a sample of 125 adults from the general population read an experimental vignette describing a young adolescent experiencing either mild or severe prodromal symptoms who was randomly assigned a ‘psychosis-risk’ or ‘high-risk reality testing’ diagnostic label, and answered questions about stigma, hope, and need for care toward the individual in the vignette. In the second study, a sample of 254 mental health professionals read the same experimental vignette who this time was randomly assigned an ‘attenuated psychosis’ or ‘reality-testing loss’ diagnostic label, and answered questions about stigma, hope, and need for care toward the individual in the vignette.

**Results:**

In the first study, the ‘high-risk reality testing’ label elicited significantly higher appraisals of self-image, hope, likelihood of seeking help, and need for care than the ‘psychosis risk’ label. Similarly, in the second study, the ‘reality-testing loss’ label elicited higher appraisals of self-image, hope, likelihood of seeking help, and importance of providing care than the ‘attenuated psychosis’ label. In both studies, no effects were found for symptom severity.

**Discussion:**

These pilot results provide first empirical support for the social and clinical potential of ‘high-risk health’ formulations in minimizing the potential stigmatizing harms of ‘at-risk’ diagnostic labels and improving help-seeking behaviors. If addition, they lay the theoretical and methodological foundation for future studies that will replicate and extend the above findings using more ecologically valid manipulations (e.g., experimental intake meeting clips) among individuals at high risk and their families.

